# Stroke knowledge and attitudes influence early hospital arrival in acute ischemic stroke: a multicenter cross-sectional survey from Hubei Province, China

**DOI:** 10.3389/fneur.2025.1669361

**Published:** 2025-09-25

**Authors:** Xiangbo Wu, Qin Zhou, Xiao Jiang, Fan Fan, Weijun Wang, Jinhua Wang, Gang Zhou, Feng Wan, Guangming Xia

**Affiliations:** Department of Neurology, Huanggang Central Hospital, Huanggang, China

**Keywords:** acute ischemic stroke, prehospital delay, stroke knowledge, stroke attitudes, China

## Abstract

**Background:**

Prehospital delay remains a major challenge in China and is a critical barrier to timely intravenous thrombolysis (IVT) in patients with acute ischemic stroke (AIS). However, the contributing factors—particularly the roles of stroke knowledge and attitudes—are not yet fully understood. This study aimed to examine the associations between stroke knowledge, attitudes, and other potential covariates with prehospital delay.

**Methods:**

We conducted a multicenter, cross-sectional study between June and December 2023 across fifteen certified stroke centers affiliated with general hospitals in Hubei Province, China. Data on sociodemographic characteristics, stroke knowledge and attitudes were collected using a structured, self-administered questionnaire. Multiple logistic regression analyses were performed to identify factors associated with prehospital delay.

**Results:**

A total of 1,870 AIS patients were included; 428 (22.9%) arrived within 3 h, and 704 (37.6%) within 6 h of symptom onset. Median prehospital delay was 11.0 h (IQR: 4.0–30.0). Multivariate logistic regression analysis revealed that higher stroke knowledge scores (adjusted OR, 1.15; 95% CI, 1.09–1.21) and higher stroke attitude scores (adjusted OR, 1.26; 95% CI, 1.18–1.33) were significantly associated with earlier arrival. Additional predictors included experiencing stroke onset at a location within 20 km of the hospital (adjusted OR, 2.50; 95% CI, 1.87–3.34), sudden progression of symptoms (adjusted OR, 2.64; 95% CI, 2.05–3.39), NIHSS score ≥6 at admission (adjusted OR, 1.34; 95% CI, 1.02–1.75), and arrival by ambulance (adjusted OR, 1.99; 95% CI, 1.48–2.68). Similar associations were observed for the 6-h threshold.

**Conclusion:**

Our findings highlight that greater stroke knowledge and positive attitudes significantly influence early hospital arrival in AIS patients. Sudden symptom onset, ambulance use, and shorter transfer distances also facilitate timely hospital arrival, whereas mild stroke contribute to delays. Thus, future stroke interventions should take these characteristics into account, which might ultimately help to promote earlier hospital arrival.

## 1 Introduction

Stroke has long been the leading cause of death in China, accounting for over 2 million deaths annually (1). Timely restoration of cerebral blood flow is critical for minimizing neurological damage following an acute ischemic stroke (AIS). Both intravenous thrombolysis (IVT) and endovascular thrombectomy (EVT) are highly time-dependent reperfusion therapies (2). According to the U.S. Centers for Disease Control and Prevention, patients should reach a hospital within 3 h of symptom onset to receive prompt treatment, which significantly improves functional outcomes (3). However, in China, most stroke patients still fail to arrive within this crucial window (3), resulting in an IVT rate much lower than that observed in high-income countries (4). In response, the Chinese government has strengthened stroke prevention and treatment efforts by expanding IVT and EVT services in hospitals and enhancing community-based stroke education (5).

Various factors influence prehospital delay, including age, place of residence, educational attainment, medical history, stroke knowledge, transportation mode, socioeconomic status, and access to healthcare services (3, 6, 7). Among these, timely recognition of stroke warning signs and the use of emergency medical services (EMS) are key to reducing delays (6, 8). Public stroke education is widely considered an effective strategy to raise awareness. Many studies have examined the impact of educational interventions on stroke knowledge and care-seeking behavior (9, 10). Although such initiatives have improved awareness of stroke symptoms, they often have limited effects on actual response behavior, suggesting that increased knowledge alone does not always translate into timely and appropriate action during a stroke episode (11, 12). Current stroke education programs largely emphasize symptom recognition but insufficiently address attitudinal factors such as urgency, treatment benefits, and perceived severity (13). This limitation may explain why greater knowledge does not consistently lead to timely hospital arrival. Therefore, investigating the role of attitudes provides a critical, yet underexplored, perspective that can enhance the effectiveness of future public health interventions.

The knowledge-attitudes-practice (KAP) model is a widely used framework in health education to examine how knowledge and attitudes shape health-related behaviors (14). This model underscores that knowledge provides the foundation for symptom recognition, while attitudes mediate or moderate whether this knowledge is translated into actual practice, such as calling EMS or seeking hospital care without delay. Without positive attitudes—such as perceiving stroke as a serious but treatable condition—knowledge alone may not be sufficient to prompt timely action (15, 16). In recent years, the application of the KAP model in stroke education has received growing attention worldwide (17, 18). However, how stroke knowledge and attitudes affect prehospital delay remains insufficiently studied. This study therefore aimed to evaluate the association between stroke knowledge, attitudes, and prehospital delay among AIS patients in China. We hypothesize that better stroke knowledge and more positive attitudes are associated with shorter delays, offering important insights for refining stroke education and prevention programs to promote timely care and improve stroke outcomes.

## 2 Methods

### 2.1 Study design

This multicenter, cross-sectional study was conducted between June and December 2023 across fifteen certified stroke centers affiliated with general hospitals in Hubei Province, China.

### 2.2 Study participants and sampling

Eligible participants were AIS patients aged ≥18 years who were admitted to hospitals within 7 days of symptom onset and consented to participate in this study, in accordance with clinical practice guidelines and existing literature (19). Exclusion criteria included transient ischemic attack, cerebral hemorrhage, in-hospital stroke, transfer from another hospital, indeterminate onset time, death shortly after admission, or ICU admission precluding interviews (8, 20). The sample size was calculated by using PASS 15.0.5 software (21) with an estimator of the percentage of patients arriving at hospitals within 3 h (11.79%) in the research followed by Yuan et al. (3), with a 95% confidence interval (CI) and 1.5% precision, yielding 1,870 participants. A cluster sampling method was used to select eligible participants. The cluster was defined as each certified stroke center of the general hospital, which allowed intravenous thrombolysis to be performed 24 h a day. Fifteen centers were randomly selected by lottery. The number of participants recruited from each stroke center was determined according to the total number of acute stroke patients treated at that center in the previous year. In each stroke center, all eligible patients admitted during the study period were consecutively included until the target sample size was reached.

### 2.3 Study variables and measurement tools

A standard-structured questionnaire (Supplementary File S1) was developed on the base of a literature review, comprising two parts as follows:

#### 2.3.1 Assessment of sociodemographic and clinical characteristics

Sociodemographic variables included age, sex, education, personal yearly income, marital status, medical insurance, residence site (urban/rural), and distance to the hospital. Medical history variables comprised previous stroke and the presence of vascular risk factors, defined as at least one of the following conditions: smoking, alcohol use, hypertension, diabetes, dyslipidemia, coronary heart disease, atrial fibrillation, obesity, or sleep disorders. Additional variables included mode of hospital arrival (by ambulance or other), progression of symptoms (sudden/gradual), and stroke severity at admission.

#### 2.3.2 Development and validation of stroke knowledge and attitude scales

Stroke knowledge was assessed with a 14-item questionnaire, adapted from previously validated instruments (22–24). These items focused on participants' recognition of common stroke warning signs (e.g., sudden numbness, difficulty speaking, headache), awareness of stroke treatment options (e.g., intravenous thrombolysis), and knowledge of appropriate emergency responses (e.g., calling EMS, going to the correct hospital department). Each correct answer was scored as 1 point, and higher total scores indicated greater stroke knowledge regarding symptoms and treatment.

Stroke attitudes were measured using a separate 6-item scale, also adapted from published literature (25). These items were designed to capture perceptions related to the seriousness of stroke, urgency of treatment, preventability, and benefits of early hospital arrival. Responses were rated on a five-point Likert scale (1 = strongly disagree to 5 = strongly agree), with higher scores reflecting a more positive attitude toward timely prehospital care.

To ensure content validity, both scales were reviewed by a panel of experts. The knowledge scale achieved index of item-objective congruence values ranging from 0.80 to 1.00, while the attitude scale yielded a content validity index from 0.82 to 1.00. Internal consistency reliability was acceptable, with Cronbach's alpha coefficients of 0.71 and 0.80, respectively.

### 2.4 Definition

The time of symptom onset was defined as the time when stroke-related symptoms first occurred. The onset-to-door time (ODT) was defined as the period from symptom onset to the earliest documented time in the emergency department or the general department of participating hospitals. The prehospital delay was divided into more than 3 h and more than 6 h of delay (26, 27). Age was categorized into young/middle-aged (<65 years) and older adults (≥65 years). Distance to hospital was defined as the straight-line distance from the location of symptom onset to the hospital and was categorized into ≤ 20 and >20 km. Stroke severity was assessed using the National Institutes of Health Stroke Scale (NIHSS); scores <6 was classified as mild stroke, and scores ≥6 as moderate-to-severe stroke (28).

### 2.5 Data collection

Sixteen trained investigators and two supervisors were recruited for data collection. The investigators, all health managers from the participating stroke centers, conducted interviews with participants, while the supervisors, experienced neurologists with over 10 years of specialization, oversaw data management. Prior to study initiation, all investigators received comprehensive training on study objectives, survey methodology, ethical considerations, and participant recruitment procedures. Following informed consent, face-to-face interviews were conducted within 72 h of admission. For patients unable to communicate, interviews were conducted with bystanders who had witnessed symptom onset and could accurately describe the arrival process. A complete English version of the interview questions was provided as Supplementary File S1. Supervisors were responsible for monitoring participant recruitment, overseeing data collection, and ensuring data integrity. The entire data collection process was rigorously supervised to maintain accuracy and reliability.

### 2.6 Statistical analysis

All statistical analyses were performed using SPSS version 23 (IBM Corp., Armonk, NY, USA). Continuous variables were described as means ± standard deviations (SD) or medians with interquartile ranges (IQR), while categorical variables were presented as frequencies and percentages. To examine baseline differences between groups (patients arriving at hospital within ≤ 3 h vs. >3 h, and ≤ 6 h vs. >6 h), we calculated standardized mean differences (SMD) for each variable. Variables with |SMD| ≥0.1 were considered potentially important covariates and included in multivariable analyses. Multivariable logistic regression analyses were then performed to identify factors independently associated with timely hospital arrival. Adjusted odds ratios (OR) with corresponding 95% confidence intervals (CIs) were reported. Statistical significance was set at a two-tailed *p*-value <0.05.

## 3 Results

### 3.1 Baseline characteristics

**Figure 1:**
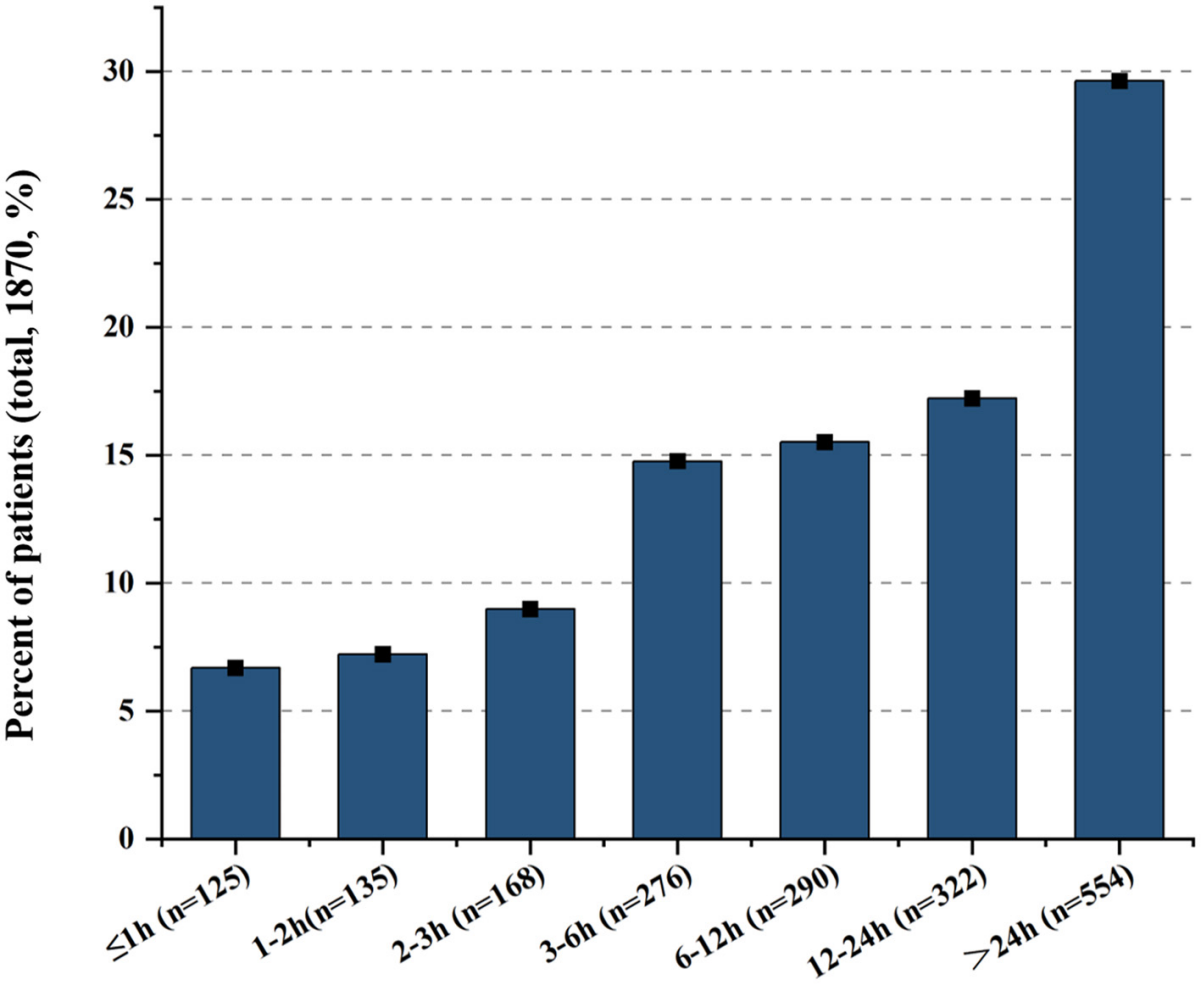
Distribution ratio of onset-to-door times in patients with acute ischemic stroke.

A total of 1,870 patients with acute ischemic stroke were recruited for this study, of whom the majority were male (61.7%) and aged ≥65 years (63.5%). Among all participants, 428 patients (22.9%) arrived at the hospital within 3 h of symptom onset, and 704 patients (37.6%) arrived within 6 h. Specifically, the proportions of patients with an ODT of 3–6 h, 6–12 h, 12–24 h, and >24 h were 14.8%, 15.5%, 17.2%, and 29.6%, respectively. Overall, 1,316 patients (70.4%) arrived within 24 h of symptom onset, whereas 554 patients (29.6%) presented after more than 24 h. The median ODT for the entire cohort was 11.0 h (interquartile range, 4.0–30.0 h; Figure 1).

**Figure 2:**
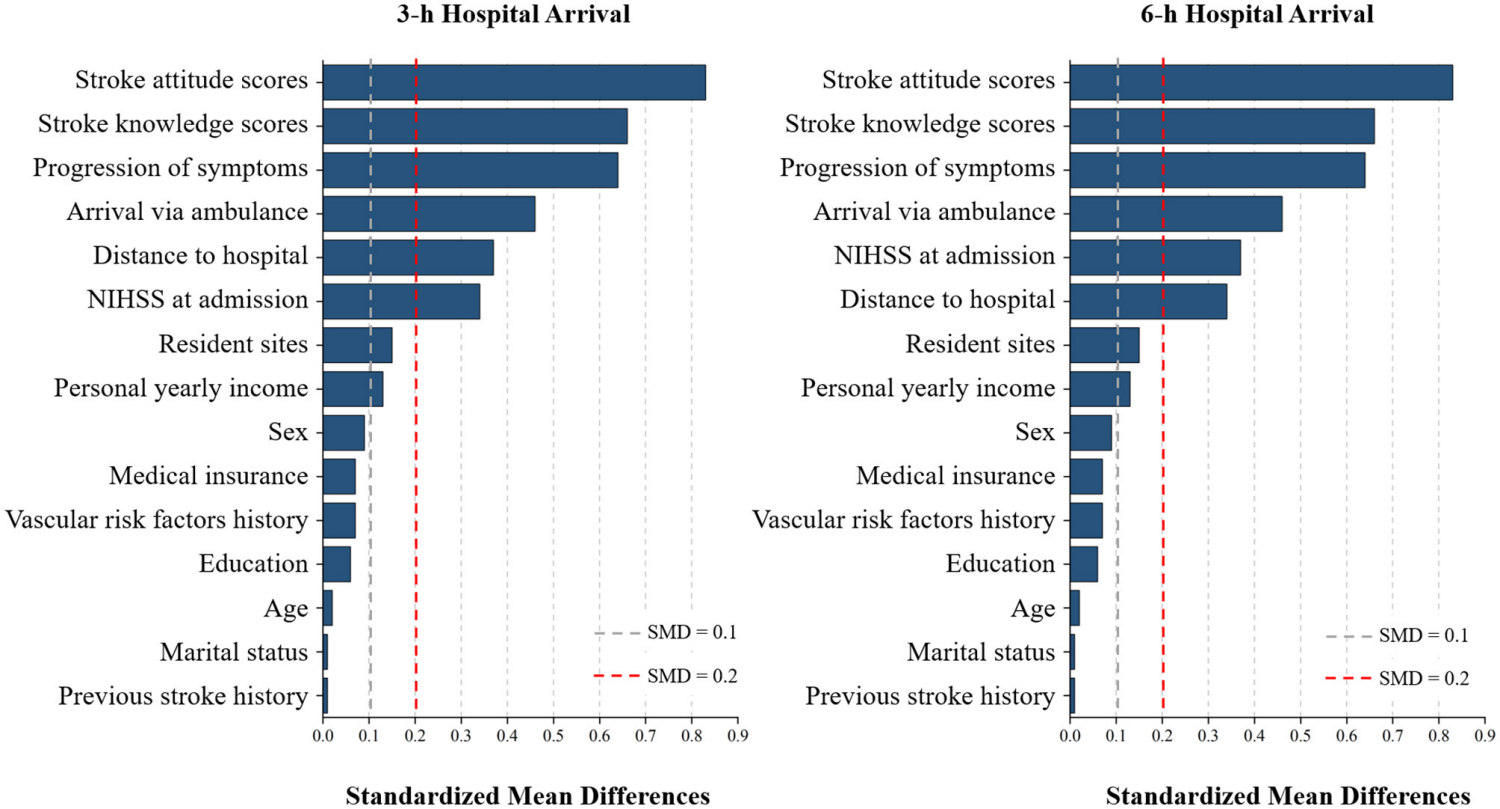
Love plots of standardized mean differences (SMD) for baseline variables related to hospital arrival times. The plots showed the standardized mean differences between patients with acute ischemic stroke arriving at hospital within 3 h **(left)** and within 6 h **(right)**. Dashed gray and red vertical lines indicate SMD thresholds of 0.1 and 0.2, representing small and medium imbalance, respectively. Variables with |SMD| ≥0.1 were considered potentially important covariates and included in multivariable logistic regression models.

As shown in Table 1 and Figure 2, patients who arrived within 3 h had notably higher mean stroke knowledge scores (7.08 ± 2.79 vs. 5.31 ± 2.66; SMD = 0.66) and stroke attitude scores (21.58 ± 2.42 vs. 19.78 ± 2.08; SMD = 0.83) compared with those arriving later. They were also more likely to present with sudden symptom onset (SMD = 0.64), arrive via ambulance (SMD = 0.46), and experience stroke onset at locations closer to the hospital (distance ≤ 20 km; SMD = 0.37). Additionally, patients arriving early were more likely to have higher NIHSS scores at admission (≥6; SMD = 0.34).

**Table 1:** Baseline characteristics in patients with acute ischemic stroke.

**Variable**	**3-h hospital arrival**		**SMD**	**6-h hospital arrival**		**SMD**
	**Yes (*****n*** = **428)**	**No (*****n*** = **1,442)**		**Yes (*****n*** = **704)**	**No (*****n*** = **1,166)**	
Age ≥65 years, *n* (%)	269 (0.63)	918 (0.64)	−0.02	443 (0.63)	744 (0.64)	−0.02
Male, *n* (%)	278 (0.65)	876 (0.61)	0.09	451 (0.64)	703 (0.60)	0.08
Marital status, married, *n* (%)	370 (0.86)	1,247 (0.86)	0.01	610 (0.87)	1,007 (0.86)	0.01
Education, Middle school or above, *n* (%)	160 (0.37)	498 (0.35)	0.06	262 (0.37)	396 (0.34)	0.07
Personal yearly income ≥5,000 CNY, *n* (%)	362 (0.85)	1,148 (0.80)	0.13	582 (0.83)	938 (0.80)	0.06
Resident sites, urban area, *n* (%)	242 (0.57)	922 (0.64)	−0.15	420 (0.60)	744 (0.64)	−0.09
Medical insurance, yes, *n* (%)	412 (0.96)	1,368 (0.95)	0.07	673 (0.96)	1,107 (0.95)	0.03
Vascular risk factors history >2, *n* (%)	156 (0.36)	476 (0.33)	0.07	252 (0.36)	380 (0.33)	0.07
Previous stroke history, *n* (%)	97 (0.23)	328 (0.23)	0.01	163 (0.23)	262 (0.22)	0.02
Distance to hospital ≤ 20 km, *n* (%)	325 (0.76)	849 (0.59)	0.37	484 (0.69)	690 (0.59)	0.20
Progression of symptoms, sudden, *n* (%)	293 (0.68)	548 (0.38)	0.64	442 (0.63)	399 (0.34)	0.60
NIHSS at admission ≥6, *n* (%)	192 (0.45)	413 (0.29)	0.34	303 (0.43)	302 (0.26)	0.37
Arrival via ambulance, *n* (%)	146 (0.34)	215 (0.15)	0.46	229 (0.33)	132 (0.11)	0.53
Stroke knowledge scores, mean ± SD	7.08 ± 2.79	5.31 ± 2.66	0.66	6.76 ± 2.77	5.08 ± 2.61	0.63
Stroke attitude scores, mean ± SD	21.58 ± 2.42	19.78 ± 2.08	0.83	21.27 ± 2.39	19.54 ± 1.96	0.81

SMD, Standardized mean differences; CNY, Chinese Yuan; NIHSS, the National Institutes of Health Stroke Scale; M±SD, Mean ± Standard deviation.

Similar trends were observed for the 6-h arrival threshold. Patients arriving within 6 h still showed higher stroke knowledge and attitude scores, were more likely to use ambulance transport, experience sudden symptom onset, and have stroke onset at locations closer to the hospital. In contrast, other sociodemographic and clinical characteristics—including age, sex, marital status, education level, personal yearly income, medical insurance coverage, previous stroke history, and vascular risk factors history—showed minimal differences between early and late arrivers, with all standardized mean differences (|SMD|) <0.1.

### 3.2 Independent factors associated with hospital arrival within 3 h

Multivariable logistic regression analysis identified several factors independently associated with hospital arrival within 3 h of symptom onset (Table 2). Higher stroke knowledge scores (adjusted OR, 1.15; 95% CI, 1.09–1.21) and higher stroke attitude scores (adjusted OR, 1.26; 95% CI, 1.18–1.33) were significantly associated with earlier arrival. Additional predictors included experiencing stroke onset at a location within 20 km of the hospital (adjusted OR, 2.50; 95% CI, 1.87–3.34), sudden progression of symptoms (adjusted OR, 2.64; 95% CI, 2.05–3.39), NIHSS score ≥6 at admission (adjusted OR, 1.34; 95% CI, 1.02–1.75), and arrival by ambulance (adjusted OR, 1.99; 95% CI, 1.48–2.68).

**Table 2:** Odds ratios and 95% confidence intervals from multinomial logistic regression for 3-h hospital arrival and 6-h hospital arrival among patients with acute ischemic stroke.

**Variable**	**3-h hospital arrival**		**6-h hospital arrival**	
	**Adjusted OR (95% CI)**	***p*** **Value**	**Adjusted OR (95% CI)**	***p*** **Value**
Urban (ref: rural)	1.21 (0.92–1.58)	0.166	–	–
Personal yearly income ≥5,000 (ref: <5,000, CNY)	1.12 (0.80–1.56)	0.514	–	–
Distance to hospital ≤ 20 (ref: >20, km)	2.50 (1.87–3.34)	<0.001	1.56 (1.24–1.96)	<0.001
Progression of symptoms (ref: gradual)	2.64 (2.05–3.39)	<0.001	2.54 (2.05–3.16)	<0.001
NIHSS at admission ≥6 (ref: <6, Scores)	1.34 (1.02–1.75)	0.033	1.45 (1.15–1.84)	0.002
Arrival via ambulance (ref: no)	1.99 (1.48–2.68)	<0.001	2.71 (2.04–3.60)	<0.001
Stroke knowledge scores	1.15 (1.09–1.21)	<0.001	1.15 (1.10–1.20)	<0.001
Stroke attitude scores	1.26 (1.18–1.33)	<0.001	1.28 (1.21–1.35)	<0.001

CNY, Chinese Yuan; NIHSS, the National Institutes of Health Stroke Scale; OR, odds ratio; CI, confidence interval.

### 3.3 Independent factors associated with hospital arrival within 6 h

Similarly, multivariable logistic regression for the 6-h threshold revealed consistent findings (Table 2). Higher stroke knowledge scores (adjusted OR, 1.15; 95% CI: 1.10–1.20) and higher stroke attitude scores (adjusted OR, 1.28; 95% CI: 1.21–1.35) remained significant predictors of timely arrival. Other independent factors included stroke onset within 20 km of the hospital (adjusted OR, 1.56; 95% CI, 1.24–1.96), sudden progression of symptoms (adjusted OR, 2.54; 95% CI: 2.05–3.16), NIHSS score ≥6 at admission (adjusted OR, 1.45; 95% CI: 1.15–1.84), and arrival by ambulance (adjusted OR, 2.71; 95% CI, 2.04–3.60).

## 4 Discussion

Our study revealed that 22.9% of patients with acute ischemic stroke arrived at the hospital within 3 h of symptom onset, with a median prehospital delay of 11.0 h (IQR, 4.0–30.0 h). Despite prehospital delay remaining a significant issue in Hubei Province, these findings nonetheless represent a notable improvement compared to earlier studies (3, 29). However, our results still lagged behind those reported in a developed region of China (30), where 33.4% of patients arrived within 3 h and the median delay was shorter at 6.0 (2.0–16.4) h. Furthermore, the timely arrival rate in our cohort remains substantially lower than figures reported in high-income countries (40%−60%) (31, 32). This improvement may be largely attributable to the broader implementation of intravenous thrombolysis programs in hospitals (5) and increased public awareness of stroke symptoms and urgency (33).

Our results indicated that greater stroke knowledge was associated with earlier hospital arrival, which is consistent with the findings of previous studies (8, 34). However, participants' recognition of specific stroke warning symptoms varied considerably. While speech disturbances and hemiplegia were commonly identified, less typical symptoms such as visual impairment and severe headache were far less recognized. Furthermore, stroke response behaviors remained inadequate: only 18.9% of participants reported that they called emergency services immediately—a proportion markedly lower than the 60.9% observed in a prior cross-sectional survey of Chinese community residents (35). This highlights a key limitation of many stroke education initiatives: improving knowledge alone does not always translate into appropriate and timely action during a stroke event (12, 35). In addition, awareness of available treatments and the urgency required for their administration is crucial to prompt decision-making and timely arrival (36). In our study, knowledge of thrombolysis was particularly poor: only 9.5% of participants knew about intravenous thrombolysis, and just 6.7% were aware of the critical time window for its use. These gaps underscore the need for future stroke education programs to focus not only on symptom recognition but also on the urgency of seeking care and understanding evidence-based treatment options.

In addition, we found that patients' or bystanders' attitudes toward stroke independently influenced early hospital arrival. Previous studies have demonstrated that attitudes—such as perceptions of disease severity, recognition of treatment urgency, and belief in the benefits of timely intervention—play a critical role in healthcare-seeking decisions and behaviors (37). A rapid response to stroke was more strongly driven by the perception that stroke is a serious and treatable condition, rather than by symptom recognition alone (38). Even when individuals correctly identified stroke warning signs, inadequate appreciation of the need for urgent treatment still contributed to prehospital delays (16). Furthermore, underestimating symptom seriousness and perceiving a low threat level were also linked to delayed hospital arrival (37). Conversely, patients who recognized the benefits of early treatment were more likely to seek prompt medical attention (8). The KAP model emphasizes the critical interplay between knowledge and attitudes in influencing stroke-related behaviors (39). While knowledge provides the foundation for symptom recognition, attitudes determine whether this knowledge is acted upon appropriately, such as promptly calling EMS or seeking hospital care (15, 16). Positive attitudes—such as perceiving stroke as a serious but treatable condition and recognizing the benefits of early treatment—are crucial for translating knowledge into timely action (16, 40). This model provides a theoretical basis for designing comprehensive stroke education strategies aimed at shortening prehospital delays.

Consistent with previous studies (16, 41), our results showed that patients who experienced sudden symptom onset were more likely to arrive at the hospital promptly. A possible explanation is that abrupt symptoms tend to draw more attention and heighten perceived disease severity compared with gradual or fluctuating symptom progression (16). Furthermore, we found that patients presenting with mild stroke were less likely to seek immediate medical care. This finding aligns with previous research (19, 42), which suggests that mild stroke often manifests with subtle or atypical symptoms that may be overlooked or misattributed to benign conditions, leading to delayed recognition and reduced urgency to seek care (26). However, even initially mild symptoms can still result in disability if not treated promptly (43). Therefore, stroke education programs should highlight the variability of stroke presentations and emphasize that all symptoms warrant urgent evaluation.

Furthermore, our findings reaffirm the critical role of emergency medical services (EMS) in reducing prehospital delay (2, 19). In this study, only 19.3% of patients arrived at the hospital by ambulance. Although this represents an improvement compared to earlier reports from Hubei Province in 2015 (15.4%) (19) and a national survey in China in 2020 (14.7%) (3), the proportion remains substantially lower than that observed in more developed regions of China (30.6%) (30) and high-income countries such as England (78.8%) (44) and the United States (51.0%) (45). We also observed notable regional disparities: patients whose stroke onset occurred within 20 km of a hospital were more likely to arrive promptly. This is consistent with previous studies (26, 46), which show that rural residents and those in economically disadvantaged areas face greater challenges to timely care—such as lower stroke awareness and limited access to emergency services (3). Addressing these disparities requires targeted health policies and improved EMS accessibility in underserved regions (3, 47).

This study has several limitations. First, its cross-sectional design allows examination of associations with prehospital delay but precludes causal inference. Second, excluding patients with very severe stroke or early death may have biased the sample toward milder cases. Because these patients often arrive earlier, our estimates of prehospital delay may be overestimated and associations with stroke severity underestimated. Third, for patients unable to communicate, attitudes were reported by proxies. As attitudes are subjective, proxy reports may not fully reflect patients' perceptions, introducing measurement error that could dilute associations. Fourth, although the knowledge and attitude scales were adapted from validated tools and showed good reliability, they were specifically developed for this study and lack external validation, which may limit generalizability. Finally, sample allocation based on patient volume at participating centers may have led to an overrepresentation of urban populations, restricting generalizability to rural settings. Despite these limitations, this multicenter survey provides comprehensive evidence that psychosocial, clinical, and healthcare system factors collectively shape prehospital delay in acute ischemic stroke. These findings highlight the need to integrate public education, EMS promotion, and improved healthcare accessibility into stroke care strategies to facilitate timely hospital arrival.

## 5 Conclusion

In summary, this multicenter study found that greater stroke knowledge, positive attitudes toward timely treatment, proximity to hospital, sudden symptom onset, and ambulance use were all independently linked to shorter prehospital delay in acute ischemic stroke. While early arrival rates have improved compared to past reports from Hubei Province, they remain lower than those in developed regions and high-income countries. These findings highlight the need for comprehensive stoke interventions to further reduce prehospital delay and improve timely access to reperfusion therapies.

## Data Availability

The original contributions presented in the study are included in the article/supplementary material, further inquiries can be directed to the corresponding authors.
