# Supplementary material for: Stroke knowledge and attitudes influence early hospital arrival in acute ischemic stroke: a multicenter cross-sectional survey from Hubei Province, China
Source: Front Neurol. 2025 Sep 25;16:1669361. doi: 10.3389/fneur.2025.1669361 (PMC12508484; doi:10.3389/fneur.2025.1669361)
Supplement: Supplementary file 1 [file Table_1.docx]

Supplementary Material

# Supplementary file 1

**Questionnaire on Prehospital Delay in Acute Ischemic Stroke**

1. Can you tell me your age? ____
2. What is your gender? (Male/Female)
3. What is your marital status? (Married/Unmarried/Divorced/Widowed)
4. What is your highest level of education?

- Illiterate or primary school (<6 years)
- Secondary school (6-9 years)
- High school (9-12 years)
- College degree or higher

1. What is your estimated yearly household income per person (CNY)?

- <5000
- 5000-20000
- 20000

1. Where do you currently reside? (Urban/Rural)
2. Do you have medical insurance? (Yes/No)
3. Do you have any of the following vascular risk factors? (Yes/No)

- Smoking (Yes/No)
- Alcohol consumption (Yes/No)
- Hypertension (Yes/No)
- Diabetes (Yes/No)
- Heart disease (Yes/No)
- Atrial fibrillation (Yes/No)
- Dyslipidemia (Yes/No)
- Sleep disorder (Yes/No)

1. Have you ever had a stroke before? (Yes/No)
2. How far was the location where your symptoms first appeared from the hospital where you first sought medical care? (≤ 20 km /＞20 km )
3. How did your symptoms appear? (Sudden/Gradual)
4. Did you arrive at the first hospital by ambulance? (Yes/No)
5. How much time passed from symptom onset to hospital arrival? (_____ hours)
6. Do you think sudden confusion or trouble speaking could indicate a stroke? (Yes/No)
7. Do you think sudden shortness of breath and difficulty breathing could indicate a stroke? (Yes/No)
8. Do you think sudden blurred vision in one or both eyes could indicate a stroke? (Yes/No)
9. Do you think a sudden severe headache without an apparent cause could indicate a stroke? (Yes/No/I don’t know)
10. Do you think sudden palpitations or chest pain could indicate a stroke? (Yes/No)
11. Do you think sudden dizziness, difficulty walking, and loss of balance could indicate a stroke? (Yes/No)
12. Do you think sudden numbness or weakness of the face, arm, or leg on one side of the body could indicate a stroke? (Yes/No)
13. After experiencing your symptoms, did you realize that you might be having a stroke? (Yes/No)
14. Are you familiar with any stroke education tools? (Yes/No)
15. Do you know about intravenous thrombolysis for ischemic stroke? (Yes/No)
16. Do you know the time window for thrombolytic therapy? (Yes/No)
17. Do you know which hospital department you should go to first when experiencing stroke symptoms? (Yes/No)
18. Do you know the first action you should take after experiencing stroke symptoms? (Yes/No)
19. Do you know how to seek emergency medical services? (Yes/No)
20. Do you believe calling emergency medical services is the most efficient way to get to the hospital after stroke onset? (Strongly disagree /Disagree /Neutral/ Agree/ Strongly agree)
21. Do you believe stroke is a serious disease that can cause disability or even death? (Strongly disagree /Disagree /Neutral/ Agree/ Strongly agree)
22. Do you believe stroke requires urgent treatment? (Strongly disagree /Disagree /Neutral/ Agree/ Strongly agree)
23. Do you think receiving hospital treatment sooner after stroke can reduce the risk of disability and death? (Strongly disagree /Disagree /Neutral/ Agree/ Strongly agree)
24. Do you believe stroke is a preventable disease? (Strongly disagree /Disagree /Neutral/ Agree/ Strongly agree)
25. Do you believe reaching the hospital within 3 hours of symptom onset could improve recovery? (Strongly disagree /Disagree /Neutral/ Agree/ Strongly agree)

(Questions for the investigator)

34. What was the patient’s NIHSS score upon admission? (_____ scores, obtained from medical records)

35. Did the patient receive intravenous thrombolytic therapy? (Yes/No, obtained from medical records)

# Supplementary Tables

**Supplementary Table 1.** Stroke knowledge among acute ischemic stroke

| **14-items** | **Correct answer rates** | |
| --- | --- | --- |
|  | **n** | **%** |
| 1. Do you think sudden confusion or trouble speaking could indicate a stroke? | 1288 | 65.7 |
| 2. Do you think sudden shortness of breath and difficulty breathing could indicate a stroke? | 895 | 47.9 |
| 3. Do you think sudden blurred vision in one or both eyes could indicate a stroke? | 660 | 35.3 |
| 4. Do you think a sudden severe headache without an apparent cause could indicate a stroke? | 632 | 33.8 |
| 5. Do you think sudden palpitations or chest pain could indicate a stroke? | 883 | 47.2 |
| 6. Do you think sudden dizziness, difficulty walking, and loss of balance could indicate a stroke? | 1123 | 60.1 |
| 7. Do you think sudden numbness or weakness of the face, arm, or leg on one side of the body could indicate a stroke? | 1416 | 75.7 |
| 8. After experiencing your symptoms, did you realize that you might be having a stroke? | 485 | 25.9 |
| 9. Are you familiar with any stroke education tools? | 246 | 13.2 |
| 10. Do you know about intravenous thrombolysis for ischemic stroke? | 177 | 9.5 |
| 11. Do you know the time window for thrombolytic therapy? | 125 | 6.7 |
| 12. Do you know which hospital department you should go to first when experiencing stroke symptoms? | 1281 | 68.5 |
| 13. Do you know the first action you should take after experiencing stroke symptoms? | 353 | 18.9 |
| 14. Do you know how to seek emergency medical services? | 1129 | 60.4 |

**Supplementary Table 2.** Stroke attitude scores among acute ischemic stroke

| **6-items** | **Mean ± SD** |
| --- | --- |
| 1. Do you believe calling emergency medical services is the most efficient way to get to the hospital after stroke onset? | 3.36 ± 0.58 |
| 2. Do you believe stroke is a serious disease that can cause disability or even death? | 3.64 ± 0.53 |
| 3. Do you believe stroke requires urgent treatment? | 3.40 ± 0.61 |
| 4. Do you believe receiving treatment sooner after stroke can reduce the risk of disability and death? | 3.18 ± 0.46 |
| 5. Do you believe stroke is a preventable disease? | 3.57 ± 0.52 |
| 6. Do you believe reaching the hospital within 3 hours of symptom onset could improve recovery? | 3.04 ± 0.53 |
